# Health Effects of Climate Change-induced Wildfires and Heatwaves

**DOI:** 10.7759/cureus.4771

**Published:** 2019-05-28

**Authors:** Michael R Rossiello, Anthony Szema

**Affiliations:** 1 Preventive Medicine, Three Village Allergy & Asthma, South Setauket, USA; 2 Allergy and Immunology, Donald and Barbara Zucker School of Medicine at Hofstra / Northwell, Stony Brook, USA

**Keywords:** wildfire, heat wave, camp fire, california, climate change, asthma, hospitalization, global warming, health, mortality

## Abstract

Global warming is a phenomenon that is affecting society in sundry ways. As of 2017, Earth’s global surface temperature increased 0.9°C compared to the average temperature in the mid-1900s. Beyond this change in temperature lies significant threats to human health in the form of natural disasters and extreme temperatures. One natural disaster that has been receiving much more attention as of 2010 is the ignition and spread of wildfires. Warmer climates lead to drier conditions, providing ideal kindling for the rapid spread of these infernos. The dangers that these intense fires pose are twofold: first, the fire causes mass property damage, physical harm, or death to the people unfortunate enough to be caught in the blaze; second, the health hazards of smoke inhalation and the emotional strain of losing one’s possessions cause immense physical and emotional harm to the fire’s victims. Another health hazard that is becoming more common due to global warming is heatwave exposure. The heat provides an ideal environment for certain pathogens to thrive, increases people’s risk of developing temperature-related health conditions, and could exacerbate many preexisting diseases. The increase in frequency and intensity of these extreme weather conditions calls for devotion of resources to fire prevention and public health measures related to smoke inhalation and heat exposure.

## Introduction and background

Climate change is a cause for concern among many people. According to the Goddard Institute for Space Studies at the National Aeronautics and Space Administration (NASA), Earth’s global surface temperature in 2017 increased by 0.9°C relative to the average temperatures between 1951 and 1980. In addition, 17 of the 18 warmest years from 1880-2017 have all occurred since 2001, with the exception of 1998 [1]. With a change in climate comes weather anomalies that can drastically affect the lives of the indigenous populations of an area. In extreme cases, these changes in global temperature can subject people to a greater risk of natural disasters. One such catastrophe that threatens the lives of people all over the world is the ignition of wildfires.

Supporting an association between global warming and wildfire frequency, duration, and devastation, a study by Westerling et al. found strong correlations between wildfire and hydroclimate in western United States (U.S.) forests, indicating that the increased wildfire activity the West Coast experienced over recent decades reflects sub-regional responses to climate change. They discovered that the frequency of wildfires from 1987 to 2003 was almost four times the average of 1970 to 1986, and that the total area burned by these fires was more than six times the previous amount. Additionally, the interannual variability in wildfire frequency was strongly associated with regional spring and summer temperature (Spearman’s correlation of 0.76, P < 0.001, n = 34). The average length of the wildfire season from 1987 to 2003 increased by 78 days, a 64% increase from the average between 1970 and 1986. This was due to a combination of earlier yearly ignitions and later dates of control. Finally, the average time between discovery and control for a wildfire increased from 7.5 days in the earlier period to 37.1 days in the later period [2]. Because wildfires are becoming more frequent and increasingly difficult to control, this amplifies the threat that they pose to human health and safety.

From 1970 to 2003, the greatest increase in wildfire frequency occurred in the Northern Rockies [2]. This supports climate change as a greater contributor to wildfire incidence than changes in human land use such as deforestation and logging. Westerling et al. determined that increases in wildfire frequency were concentrated between elevations of 1680 to 2590 meters and were episodic, with surges in warm years and relatively little activity in cool years. Also, they discovered the existence of a strong association of wildfire activity with changes in spring snowmelt timing, which is sensitive to temperature change. Within the period of study, 56% of wildfires and 72% of area burned in wildfires occurred in early snowmelt years, while 11% of wildfires and 4% of area burned occurred in late snowmelt years [2]. As temperatures rise earlier in the year, the soil dries out sooner and the flora become more flammable as water from winter precipitation is extracted from them. An increase in global temperatures and a drier atmosphere in the West increases the likelihood of sparking more devastating fires with far greater destructive potential.

## Review

The costs

The increase in frequency and duration of wildfires places a large financial burden on federal and state governments. According to the National Interagency Fire Center, the U.S. Forest Service’s annual suppression costs have exceeded one billion dollars for 13 of the 18 years between 2000 and 2017 [3]. This is a significant increase in average annual costs from those between the years of 1985 and 1999, during which the total annual suppression costs never once exceeded one billion dollars.

Governing bodies are not the only ones who suffer immense costs as a result of a wildfire. These catastrophes take a far greater toll on their victims, who often lose their entire livelihood, and sometimes their lives, in the blaze. In the course of just a few hours, people can lose their homes, businesses, pets, family heirlooms, and sentimental items that hold far more value to them than to insurance companies. Victims only recover approximately ten percent of their financial losses and are allotted a maximum of 2-2.5 years to complete their projects and close out their claims. This places an extra burden on them as they navigate a rebuilding process riddled with hurdles to overcome and permissions to obtain and ensure that their new home complies with updated building codes and regulations [4]. Although victimized communities make efforts to update infrastructure and safety regulations and use more flame-retardant materials, the fact remains that another wildfire could be ravaging through their region in the not-so-distant future. Many of these victims must decide whether they want to cut their losses and leave or undergo the rebuilding process and the risks that come with it. Many of these victims are displaced from their homes during the rebuilding period, leading to congestion in neighboring communities, placing a large strain on their resources. According to CAL FIRE, the Department of Forestry and Fire Protection in the state of California, 15 of the top 20 most destructive California Wildfires occurred between the years 2000 and 2018, with 10 of those fires occurring within the past five years [5]. This statistic is concerning, not only for the safety of the residents of California but for their health as well. One speculation is that recent overbuilding in previously rural regions has led to greater risk for fires.

Health hazards

Wildfire smoke contains many air pollutants of concern for public health, such as carbon monoxide (CO), nitrogen dioxide (NO_2_), ozone (O_3_), particulate matter (PM), polycyclic aromatic hydrocarbons (PAHs), and volatile organic compounds (VOCs). Exposure to wildfire smoke has also been associated with an increase in mortality rate. A 13.5-year study that included 48 days of wildfire smoke in Sydney, Australia demonstrated a significant increase in mortality associated with the smoke-affected days. Additionally, a meta-analysis of data from 2003 to 2010 in 10 cities in Europe found increases in cardiovascular mortality associated with PM_10_ levels that were stronger on smoke-affected days than on non-affected days [6]. In a 2012 study, Johnston et al. estimated the annual global premature mortality rate due to wildfire smoke exposure to be 339,000 deaths [7].

In addition to increases in mortality, wildfire smoke exposure has also been associated with respiratory distress and declines in lung function among non-asthmatic children. People exposed to wildfire smoke have reported an increase in visits to their physicians for respiratory issues, an increase in respiratory emergency department visits, and respiratory hospitalizations. Increasing amounts of evidence for associations between wildfire smoke and chronic obstructive pulmonary disease (COPD), acute bronchitis, and pneumonia have also been discovered [6].

Numerous epidemiological studies show that exposure to wildfire smoke causes exacerbations of asthma. Interestingly, some studies demonstrated no significant acute changes in lung function among people with asthma related to PM from wildfires. However, they did report significant declines in lung function among exposed people without asthma and children without bronchial hyper-reactivity [6]. This may be due to the use of respiratory medications by asthmatics and the lack of medication use by non-asthmatics. Other studies highlighted an association between medication use for obstructive lung disease and wildfire smoke exposure. There was an increase in both the usage of reliever medications and initiation of oral steroid use associated with wildfire smoke [6]. Another study by Tse et al. documented increases in physician-dispensed short-acting beta-agonists but not physician-prescribed oral corticosteroids for children with asthma in the years following two catastrophic wildfires in Southern California [8].

Camp Fire

**Figure 1:**
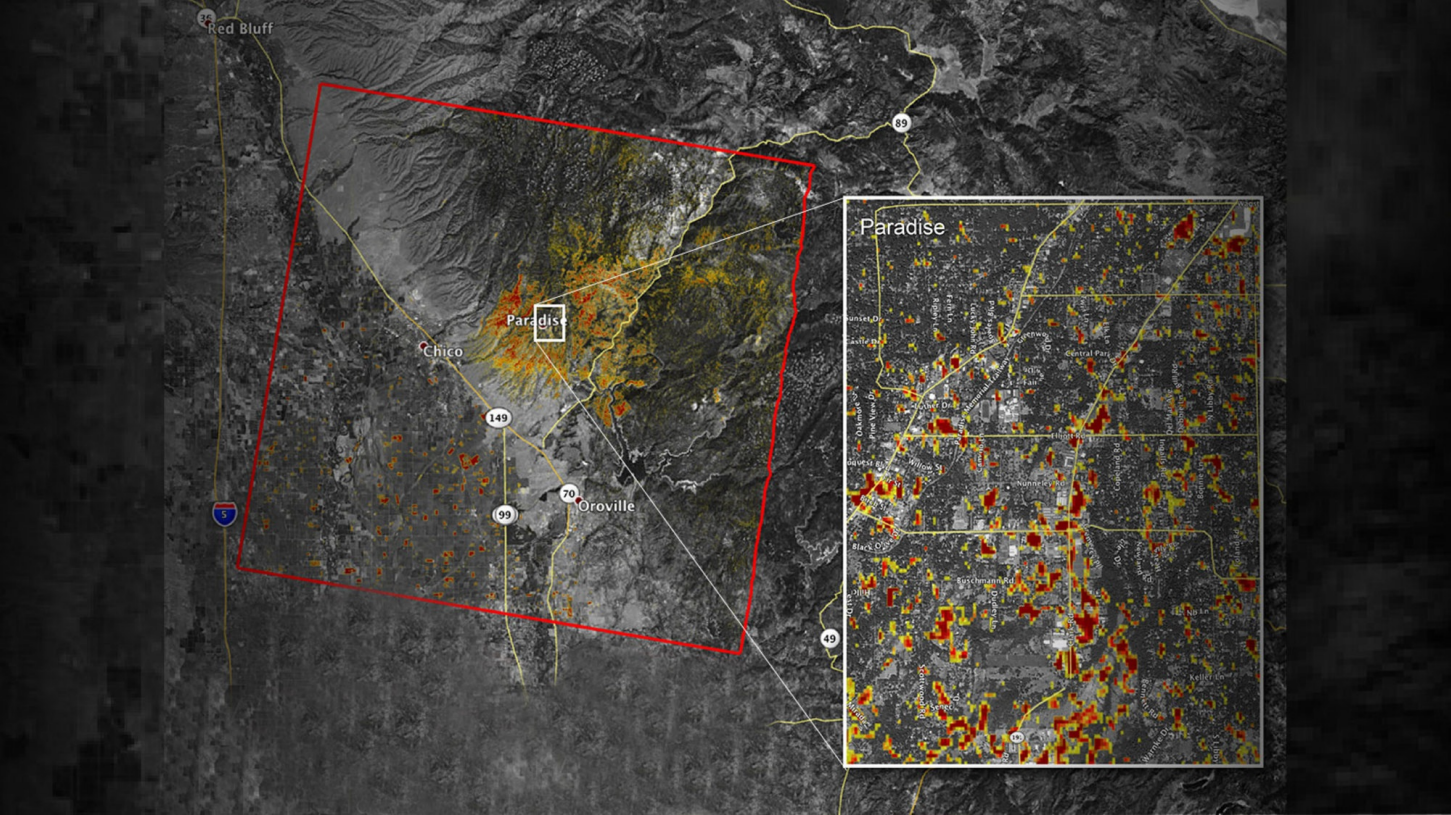
Satellite image of the damage to Paradise, California from the Camp Fire as of November 16, 2018. "The map was developed using synthetic aperture radar images from the Copernicus Sentinel-1 satellites operated by the European Space Agency. The map covers an area of 48 miles by 48 miles (78 by 77 kilometers), outlined in red on left. A closeup view of damage to the town of Paradise is inset on right, outlined in white. The color variation from yellow to red indicates increasingly more significant changes in the ground surface." Courtesy NASA/Jet Propulsion Laboratory-California Institute of Technology (JPL-CalTech). https://www.jpl.nasa.gov/news/news.php?feature=7278. Accessed 02/15/2019

Beginning on November 8, 2018, in the Sierra Nevada foothills, the Camp Fire raged throughout Northern California’s Butte County for 17 days, before being one hundred percent contained by firefighters [9]. It was the most destructive fire in California’s history, burning over 153,000 acres of land and incinerating nearly 19,000 structures. Eighty-six deaths were reported as a result of the blaze, making the Camp Fire the deadliest wildfire in the state of California. The death toll is nearly four times that of the Tubbs Fire, which is now the second most destructive fire in California history [5]. One of the most affected communities was the town of Paradise, which was totally annihilated in the fire (Figure 1). Few structures managed to survive the inferno; what remained was uninhabitable. Furthermore, the temporary accommodations for victims of the Camp Fire were inundated by overcrowding. More than 120 people were sickened by an infection with norovirus [9].

Woolsey Fire

**Figure 2:**
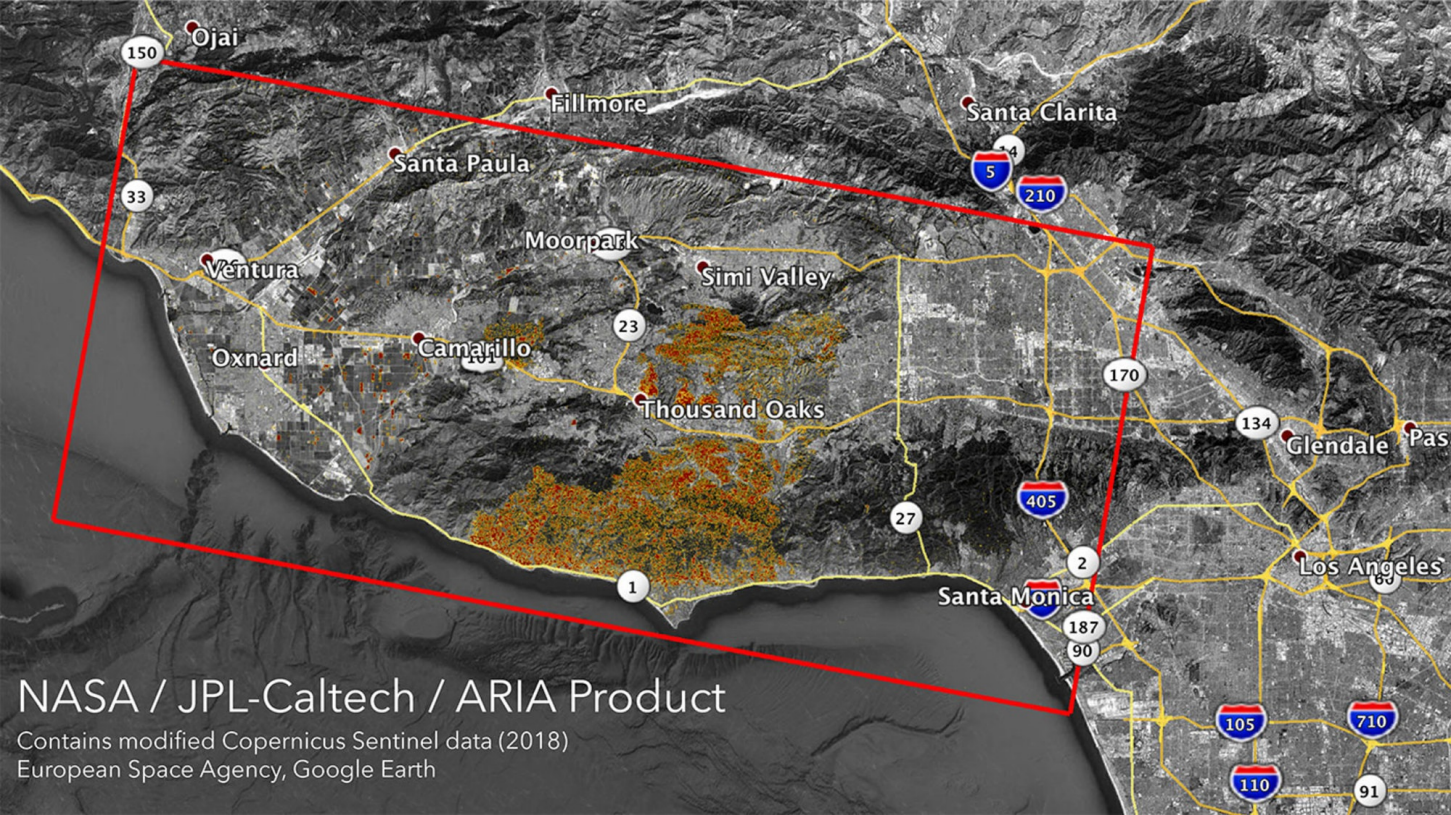
Satellite image of the damage to Southern California from the Woolsey Fire as of November 11, 2018. "Areas likely damaged by the Woolsey Fire as of Sunday, Nov. 11. It covers an area of about 50 miles by 25 miles (80 km by 40 km) — framed by the red polygon. The color variation from yellow to red indicates increasing ground surface change, or damage." Courtesy NASA/JPL-Caltech.https://www.jpl.nasa.gov/news/news.php?feature=7278. Accessed 02/15/2019

Occurring simultaneously with the Camp Fire was the Woolsey Fire. This wildfire ignited on the site of the old Santa Susana test lab near Simi Valley in Ventura County on November 8, 2018 [10]. The fire grew to be one of California’s most destructive wildfires, incinerating nearly 97,000 acres of land and over 1,600 structures. The inferno also claimed three lives [5]. The rapid growth of the fire was attributed to strong winds - which accelerated the spread of the flames - and a lack of containment resources. Rescue workers in Southern California had their hands full battling the nearby Hill Fire in Santa Rosa Valley, placing a strain on firefighting personnel and resources. The *Los Angeles Times* reported that by 7:30 p.m. on November 8, 400 personnel were battling the Hill Fire while only 150 firefighters from three agencies were attempting to control the Woolsey Fire [10]. The fire rapidly made its way south into Los Angeles County, ravaging parts of Thousand Oaks and Malibu (Figure 2).

Heatwaves

Another consequence of global warming is the increase in frequency of heatwaves. Heatwaves not only contribute to the drying of the land and a subsequent creation of fuel for the rapid spread of wildfires, but also increase the mortality rate and the frequency of hospitalizations in the areas they affect. This is especially problematic when a heatwave hits an area with a normally cool climate because the residents are not accustomed to the intense heat. A review by Rossati on the health impacts of global warming states that mortality generally increases at temperatures above and below optimum value. For example, a heatwave in Europe during August of 2003 resulted in an excess mortality of 14,800 deaths. Additionally, people with preexisting, chronic conditions such as hypertension, heart disease, diabetes, and obesity are more vulnerable to the heat and at risk of complications. Asthma patients were hospitalized more frequently during the extreme weather period, and the risk of out-of-hospital cardiac arrest was reported to rise by 14%. Extreme heat is also known to increase the incidence of urolithiasis [11].

A study examining heatwaves in California from 1999 to 2009 found that hospital admissions increased by seven percent on the peak heat-wave day. These investigators observed a significant impact on cardiovascular disease, respiratory disease, dehydration, acute renal failure, heat illness, and mental health. Over the period of study, 11,000 excess hospitalizations due to extreme heat were observed [12].

## Conclusions

In conclusion, wildfires are increasing in prevalence, largely as a result of climate change. Higher temperatures have dried vegetation to produce fuel and concurrent drying of groundwater accelerates combustion. Physicians should note the impact of global warming on human health with regard to fires and heatwaves so they can be attuned to relevant medical conditions and can proactively help their patients.
